# Lactosuria

**Published:** 1887-03

**Authors:** 


LACTOSURIA.—Dr. A. B. Leone defines lactosuria as the elimination by the kidneys of milk sugar formed in the breasts. The milk not being used to nourish the infant, becomes re-absorbed in the blood vessels and lymphatics then entering the circulation whence it is excreted by the kidneys. Lactosuria is not observed before the seventh month of pregnancy, and is not constant in the last two months, but depends upon the development of the breasts.

In women who do not nurse their children, lactosuria is quite constant in the first five or six days of the puerperium, but is sometimes absent the first day. In the second day it is constant, but the lactose may be in small quantities. It increases on the third and fourth days, and then declines.

In nursing women, the phenomenon is observed during the first days after labor, the quantity of lactose varying in quantities from one to three grammes per litre. If women stop nursing for any cause, lactosuria is promptly developed, but disappears within a few days.

Lactosuria during the first days of the puerperium, and of women who do not nurse, disappears during fever.—*Rivista Chir. e Terap.*

